# Cell-Free Fetal DNA and Non-Invasive Prenatal Diagnosis of Chromosomopathies and Pediatric Monogenic Diseases: A Critical Appraisal and Medicolegal Remarks

**DOI:** 10.3390/jpm13010001

**Published:** 2022-12-20

**Authors:** Giuseppe Gullo, Marco Scaglione, Giovanni Buzzaccarini, Antonio Simone Laganà, Giuseppe Basile, Vito Chiantera, Gaspare Cucinella, Simona Zaami

**Affiliations:** 1 Department of Obstetrics and Gynecology, Villa Sofia Cervello Hospital, IVF Unit, University of Palermo, 90133 Palermo, Italy; 2 Department of Neuroscience, Rehabilitation, Ophthalmology, Genetics and Maternal-Child Sciences, University of Genoa, 16132 Genoa, Italy; 3 Department of Women’s and Children’s Health, Gynaecologic and Obstetrics Clinic, University of Padua, 35122 Padua, Italy; 4 Unit of Gynecologic Oncology, ARNAS “Civico—Di Cristina—Benfratelli”, Department of Health Promotion, Mother and Child Care, Internal Medicine and Medical Specialties (PROMISE), University of Palermo, 90133 Palermo, Italy; 5 IRCCS Orthopedic Institute Galeazzi, 20161 Milan, Italy; 6 Department of Anatomical, Histological, Forensic and Orthopedic Sciences, “Sapienza” University of Rome, 00161 Rome, Italy

**Keywords:** NIPT, prenatal diagnosis, chromosomopathies, cell-free DNA, fetal DNA, medicolegal traits

## Abstract

Cell-free fetal DNA (cffDNA) analysis is a non-invasive prenatal diagnostic test with a fundamental role for the screening of chromosomic or monogenic pathologies of the fetus. Its administration is performed by fetal DNA detection in the mother’s blood from the fourth week of gestation. Given the great interest regarding its validation as a diagnostic tool, the authors have set out to undertake a critical appraisal based on a wide-ranging narrative review of 45 total studies centered around such techniques. Both chromosomopathies and monogenic diseases were taken into account and systematically discussed and elucidated. Not surprisingly, cell-free fetal DNA analysis for screening purposes is already rather well-established. At the same time, considerable interest in its diagnostic value has emerged from this literature review, which recommends the elaboration of appropriate validation studies, as well as a broad discourse, involving all stakeholders, to address the legal and ethical complexities that such techniques entail.

## 1. Introduction

Since its discovery in 1997, cell-free fetal DNA (cffDNA) has represented a milestone in non-invasive prenatal diagnostics. Mixed with maternal cell-free DNA, from which it cannot be separated completely, cffDNA can be found from the fourth week of gestation in amounts ranging between 5 and 20%. From a technical standpoint, it is certainly difficult to perform, first of all because of the small amount of genetic material present in the sample if compared to the maternal one. Its use in the prenatal diagnosis of monogenic transmission diseases was described for the first time in 2000. Since 2012, screening for several diseases has been available in the UK healthcare system on request. The identification of maternally inherited genetic variants is based on dosage-based techniques that detect small differences in the levels of mutant and wild-type alleles. In contrast, for de novo and paternally inherited variants, it is necessary to rely on techniques such as next-generation sequencing (NGS) and digital polymerase chain reaction (PCR) to detect variants in the cffDNA that are not present in the maternal cfDNA [1]. As far as monogenic pathologies are concerned, among the most widespread techniques we find the relative haplotype dosage approach, which has the advantage of simultaneously identifying both the variants inherited paternally and maternally (measuring the haplotype dosage imbalance in maternal plasma DNA). The technique has been successfully used for Duchenne muscular dystrophy, congenital adrenal hyperplasia, maple syrup urine disease, hyperphenylalaninemia and spinal muscular atrophy. Among the various sub-methods reported by scientific sources, it is worth mentioning approaches such as clone pool dilution sequencing, contiguity-preserving transposition sequencing, targeted locus amplification (TLA), HaploSeq and long fragment read (LFR) technology. Such techniques entail long and complex experimental phases, and often present low success rates when used separately. These limitations can be overcome by the population-based method, based on reference population with genotyping data of unrelated individuals, which achieves 80% accuracy in non-invasive prenatal diagnosis. In order to further improve the success rate and accuracy of haplotype phasing, some authors have experimented with the combination of different methods [2]. The progressive technological advancement has allowed an increasing diffusion of these screening tests, which are now widely consolidated for prenatal diagnosis of chromosomal diseases, in particular Down, Edwards and Patau Syndrome. It should be emphasized that, at the moment, the non-invasive prenatal test (NIPT) remains a screening test and, for this reason, requires confirmation with the invasive method. In the Italian national health system, the screening test for chromosomopathies is available on payment, for all families. At the moment it is still premature to hypothesize an inclusion of the test in the essential levels of care (mainly for understandable bioethical issues arising from the difficulty of choosing to whom to provide priority testing) [3]. However, it is essential to understand the potential of a test that, for some diseases, can allow an even earlier diagnosis than neonatal screening, leading to adequate management of complications and, in some cases, also of therapy. The purpose of this review is to show some studies that support the efficacy of NIPT for aneuploidies and, secondly, to provide a complete picture of the use in recent years as regards the prenatal diagnosis of different monogenic diseases, mainly starting in pediatric age. Moreover, this review is aimed at laying the groundwork for possible future validation of cffDNA diagnostic role.

## 2. Materials and Methods

### 2.1. Study Design

This is a qualitative review centered around non-invasive prenatal diagnosis, with a close focus on chromosomopathies and monogenic transmission disorders.

The review has been rationally divided it into three sections. The first one elaborates on studies concerning cell-free DNA non-invasive prenatal diagnosis of the most common aneuploidies (21, 18 and 13 Trisomies); the schematization of such studies was focused on the performance in terms of diagnostic accuracy. Although studies on twin pregnancies are limited, relevant data from the existing ones have been reported. Since data on sex chromosome aneuploidies were not reported by all authors, they were not considered when reporting the results. The second section was devoted to the analysis of studies that reported non-invasive prenatal diagnosis analysis with cell-free fetal DNA for copy number variation (CNV)-related conditions. The third section draws upon sources elaborating on the use of cffDNA analysis for non-invasive prenatal diagnosis (NIPD) of monogenic transmission disorders; the discussion of such studies has in turn been subdivided according to the nosological category of treated diseases.

### 2.2. Eligibility Criteria

All papers evaluating the cell-free DNA non-invasive prenatal diagnosis have been included, irrespective of study design and date of publication, from 2011 onward, in order to ensure a high degree of reliability and updated sources. The inclusion criteria were as follows:

Language: studies written in English.
Study design: no restriction.
We excluded from this review any source exhibiting insufficient data to elaborate and report results.

### 2.3. Information Sources and Search Strategy

Electronic databases (ScienceDirect, MEDLINE, Scopus, Embase, the Cochrane Library, Clinicaltrials.gov, EU Clinical Trials Register, and the World Health Organization International Clinical Trials Registry) were searched until April 3, 2022. We used the following key words: cell-free DNA [mesh] AND prenatal OR fetal AND diagnosis AND NIPT AND (non-invasive OR DNA). A manual search of the reference lists for all included studies and review articles was then carried out in order to detect missed papers. We searched for published (full-text studies and meeting abstracts) and unpublished studies or “grey literature” (i.e., for which only a registered protocol was available) from the aforementioned electronic databases.

### 2.4. Study Selection and Data Extraction

Three authors (GB, MS and ASL) independently screened the titles and abstracts of the selected papers. The text of each potentially relevant study was considered for inclusion in each section of the review independently by two authors (GB and MS). They also independently extracted data from the included studies. Another author (GG) independently reviewed the selection and data extraction process. The results were compared, and any disagreements were discussed and resolved by consensus. For the three categories of pathologies presented, only studies in which a population with non-invasive prenatal diagnosis was reported, confirmed with another method in the pre- or post-natal period, were included. In so doing, the authors aimed to provide a clear and rather comprehensive picture as to the different levels of reliability in each method, based on the individual condition considered.

## 3. Results

**Figure 1:**
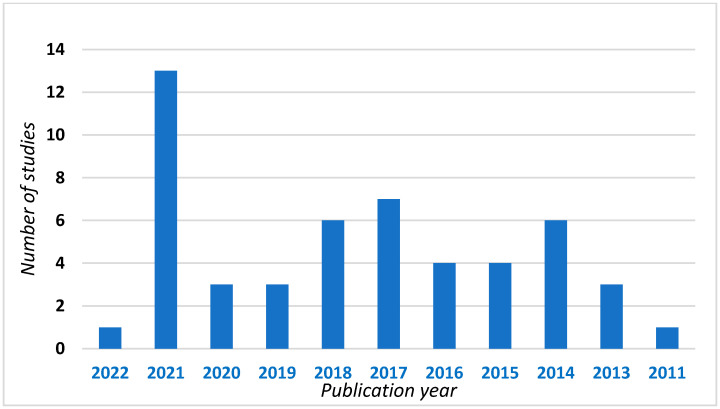
Studies included in this review divided according to year of publication.

A total of 51 studies were ultimately included in this review. Figure 1 shows the partition of the reviewed studies according to year of publication. We can see that most of them were published in 2021 (13 out of 51 studies), likely due to the relatively young age of the investigation technique. Regarding the reviewed studies, 13 are described in the first section concerning the role of cffDNA in the non-invasive prenatal diagnosis of aneuploidies [4,5,6,7,8,9,10,11,12,13,14,15,16], 7 are described in the second section (CNV diseases) and the remaining 31 are in the third section concerning diseases with monogenic transmission [2,17,18,19,20,21,22,23,24,25,26,27,28,29,30,31,32,33,34,35,36,37,38,39,40,41,42,43,44,45,46].

### 3.1. Non-Invasive Prenatal Diagnosis of Chromosomal Aneuploidies

Table 1 shows the detection rates for the most common trisomies compatible with life (i.e., Down syndrome, Edwards and Patau). Looking at the median values reported we can see that the performance of the test is high for all three diseases (99.50%, 99.12%, 99.99%, respectively). This justifies the use of cffDNA as a screening tool for these chromosomal disorders NIPD.

Considering that the introduction in the clinical setting is dated around 2011, already in 2013 Liang et al. [15] described the feasibility of non-invasive prenatal diagnosis on plasma samples from 435 women with high-risk pregnancy for Down syndrome, thus before amniocentesis. A sequencing at low coverage was performed and the results were compared with the karyotype (of the samples, 94.7% had karyotype and complete sequencing results). Fetal aneuploidies such as trisomy 21, trisomy 18, trisomy 13 and trisomy 9 were accurately identified with a detection sensitivity of 100% and a detection specificity of 99.71% [4].

Lau et al. in 2014 [13] reviewed the results of a non-invasive prenatal test of 1982 pregnancies, again based on low coverage whole-genome sequencing of maternal plasma DNA. NIPT was positive for common trisomies in 29 cases, all were confirmed by prenatal karyotyping (specificity = 100%). The clinical outcome was evaluated in 85.15% of patients. Three chromosomal abnormalities were not detected by NIPT, including one case each of a balanced translocation, unbalanced translocation and triploidy. There were no known false negatives involving the common trisomies (sensitivity = 100%). These data confirm that already in the last decade the high diagnostic accuracy of the non-invasive test was known, certainly for the most common trisomies [13].

Porreco et al. [12], in a cohort study published in 2014, reported the results of a massively parallel genomic sequencing of cffDNA contained in specimens from 3430 pregnant women at high risk for fetal aneuploidy. In this case series there were no false-negative results for trisomy 21 while there were three for trisomy 18, and two for trisomy 13, indicating a lower sensitivity. All three false-positive results were for trisomy 21, indicating a lower specificity for this disease. However, the positive predictive values for trisomy 18 and 13 were 100% and 97.9% for trisomy 21. 

Zhang et al. [11] in 2015 reported on a large case series (146,958 samples studied always with low-coverage whole-genome sequencing of plasma cell-free DNA) that there was no significant difference in test performance between the high-risk and low-risk subjects (sensitivity, 99.21% vs. 98.97%, *p* = 0.82; specificity, 99.95% vs. 99.95%, *p* = 0.98), further validating its efficacy.

A 2016 review by Taylor-Phillips et al. [10] reported high sensitivity rates for trisomy 21, slightly lower for 18 and 13. It is, however, stressed by the authors that although precise, the diagnostic accuracy of the test never reaches 100% and for this reason, limits the use of this test for diagnostic (but not for screening) purposes.

In a 2018 paper [8], Miltoft et al. reported how, in a clinical setting with efficient combined first trimester screening (cFTS, characterized by the nuchal translucency thickness and levels of pregnancy-associated plasma protein A (PAPP-A) and β-human chorionic gonadotropin (β-hCG)), contingent screening offering women with a cFTS risk of ≥1 in 100 an invasive test and women with a risk from 1 in 100 to 1 in 1000 a cfDNA test had the same sensitivity for T21, T18 and T13, but significantly increased specificity, when compared with offering an invasive test to all women with a risk of ≥1 in 300. This indicates how improved conditional screening protocols could lead to less use of invasive techniques. A 2020 cohort study by Serapinas et al. [6] reported that NIPT (performed by a single nucleotide polymorphism method) achieved a high positive-predictive value (PPV) for both trisomy 21 and 18. Such a finding confirms the possible use of this test as a definitive diagnostic tool, certainly with regard to trisomy 21. Importantly, in this case series a significant difference was found between the fetal fraction of samples belonging to the no-call group (3.1%) and that of samples whose patients received a call (9.1%). The above parameter was found with positive correlation to gestational age.

Bardi et al. 2020 [16] reported that, although cffDNA is superior to the combined test, especially for the detection of trisomy 21, about one of three congenital abnormalities may remain undetected in the first trimester of pregnancy, unless the cfDNA test is used in combination with fetal sonographic study, including NT measurement.

A 2021 cohort study by Borth et al. [5] reported high sensitivity and specificity of the test (of ≥98.89%) for all three trisomies. Interestingly, in this case study the accuracy of the test was validated with a careful clinical follow-up performed after birth.

Of the studies under review, two focused on the performance of the test in the setting of twin pregnancies. In a 2021 study [4], Judah et al. reported a very good performance with regard to trisomy 21, albeit still lower when compared to singleton pregnancies. In this study, although high detection rates are reported also for the other two chromosomal diseases, the authors report the data as small in order to make definitive considerations. In contrast, a 2019 cohort study by Gil et al. [7] reported totally comparable test sensitivity rates between twin and single pregnancies for trisomy 21. For trisomy 18 and 13, even in this cohort, given the lower incidence of these conditions, insufficient data are reported to express with certainty on the real performance of the test.

### 3.2. Non-Invasive Prenatal Diagnosis of CNV Diseases

Table 2 summarizes the series in which NIPD of CNV pathologies was presented.

Among reviewed studies, two reported applications of NIPD for diseases such as Angelman syndrome, Prader-Willi, Di George, Cri du Chat, and from 22q11.22 microduplication [50,53]. Angelman and Prader-Willi (PW) syndromes are diseases characterized by different alterations at the chromosome 15 (maternal uniparental disomy leads to PW and paternal to Angelman syndrome). In particular, Prader-Willi syndrome is characterized by different clinical signs depending on the age of life (poor fetal movements in pregnancy; generalized hypotonia and difficulty in nutrition in the first years of life; hyperphagia and disturbances of different endocrine systems in growth with variable degree of cognitive deficits). Di George syndrome causes thymic hypoplasia with immune deficiency, parathyroid hypoplasia (resulting in hypocalcemia), facial mass malformations, congenital heart malformations, and variable degree of cognitive deficits. According to the 2017 article by Petersen et al. [53], in which the genetic material has been analyzed with NGS method, for the microdeletion syndrome regions a reduced accuracy of the test has emerged in terms of positive predictive values (0% for detection of Cri-du-Chat syndrome and Prader-Willi/Angelman syndrome; 14% for 1p36 deletion syndrome and 21% for 22q11.2 deletion syndrome) with high false-positive rates. These results, according to the authors, are related to the low prevalence of these syndromes in the general population. Such a hypothesis seems to be supported by the study of Liang et al. in 2019 [50], which involved a large cohort of 94,085 of pregnant women and found an improvement of PPVs (93% for DiGeorge, 68% for 22q11.22 microduplication, 75% for Prader-Willi/Angleman; 50% for Cri du Chat). Other authors have discussed the application of non-invasive prenatal diagnosis with respect to other CNVs. Out of a large series of more than 18,000 patients, Yunsheng et al. in 2021 reported that the accuracy of the test is still relatively low and needs improvement [49]. On the contrary, Kaseniit et al., in a 2018 study, reported how, with proper algorithm design and extensive testing, NIPT can achieve high specificity also for CNV alterations [52]. Songchan et al., in a 2021 study, highlight how an adequate analysis as an NGS method, can be very valid as long as the depth of reading is adequate; this in fact allows us to reduce the percentage of false positives and consequently increase the specificity of the test [48].

### 3.3. Non-Invasive Prenatal Diagnosis of Monogenic Transmission Diseases

Table 3 reports the studies in which a case history of NIPD of monogenic transmission pathologies was presented.

The table also includes conditions resulting from chromosomal microdeletions/duplications, reported in this section as not classifiable in the aneuploidies described in the previous paragraph. Although most of the diseases described are transmitted by autosomal recessive mode, autosomal dominant and X-linked inheritance disorders are also described.

**Figure 2:**
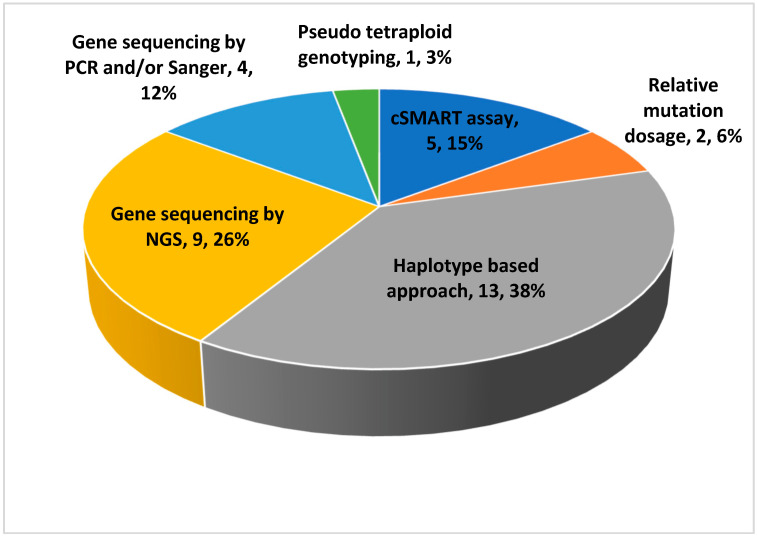
Subdivision of the studies based on the analytical methods used.

Moreover, Figure 2 shows the subdivision of the studies based on the methods of analysis used. Of them, the haplotype-based approach is the most used, as it is a method that allows the simultaneous identification of variants inherited as paternally, maternally, and de novo. In almost all studies, the pregnancies belonged to families at risk for that particular disease and the result of the non-invasive method was compared with the result of genetic testing obtained with invasive method. The discussion of the various studies was divided into sub-sections according to the nosological category.

#### 3.3.1. Endocrine System and Bone Diseases

Congenital adrenal hyperplasia (CAH) is the most cited pathology in the field of non-invasive prenatal diagnosis, probably because of the possibility of treatment already in gestational age. In the affected child we basically recognize two forms, classical and non-classical, determined by different genetic mutations and consequently by a different functionality of 21-alpha hydroxylase, an enzyme whose malfunction is responsible for over 90% of cases of CAH. The non-classical form can remain unrecognized until the time of pubertal development (not infrequently causing a mixed-type precocious puberty) and can give clinical signs of hyperandrogenism that in young women enter in differential diagnosis with polycystic ovary syndrome. The classic form has manifestation already in young children and we distinguish a simple virilizing form and a salt wasting form. The salt wasting form can occur as early as the first months of life and, in case of failure to recognize (more frequent in males than in females), can lead to fatal consequences such as acute adrenal crisis, characterized by hypotension and hyperkalemia. It has been shown that fetal hyperandrogenism and genital ambiguity is preventable with low-dose dexamethasone initiated before the 9th week of gestation. In seven of eight at-risk pregnancies, the unaffected fetus is unnecessarily exposed to dexamethasone for weeks until the diagnosis of CAH is done by invasive procedures. It is therefore important to find a valid method of non-invasive prenatal diagnosis that can reserve the administration of the drug only to affected fetuses (exclusively female) [43,44].

A 2014 study by New et al. [39] assessed 14 pregnancies from at-risk families for the presence of CYP21A2 gene mutations by parental haplotype study. In all 14 families, the fetal CAH status was accurately extrapolated by targeted massively parallel sequencing (MPS) of DNA in maternal plasma, as early as 5 weeks 6 days of gestation (among these, only one female fetus was treated). Ma et al., in a study published in the same year [38], have successfully diagnosed the pathology in an affected fetus; with the assistance of the parental haplotypes, fetal haplotypes were recovered using a hidden Markov model through maternal plasma DNA sequencing by a similar method. The result was then confirmed by the invasive method. The test showed an accuracy of 96.41% for the inferred maternal alleles and an accuracy of 97.81% for the inferred paternal alleles. The same author, in 2017, successfully determined the CYP21A2 genotype on the 14 plasma samples from 12 families at risk, as early as day 1 at 8 weeks of gestation [32].

Among the bone pathologies for skeletal dysplasia we find several cases of NIPD. As shown in Table 3, we find achondroplasia, osteogenesis imperfecta, and thanatophoric dysplasia. Although for this group of pathologies no treatment is foreseen in the gestational period, an early diagnosis would lead to an adequate management of the more frequent complications, such as early recurrent fractures in osteogenesis imperfecta or stenosis of the foramen magnum in achondroplasia; in some patients these problems are already present from the first months of life. Moreover, in these diseases the ultrasonographic diagnosis is often inaccurate and late. In all the cited sources [21,26,34] a correct identification of the mutation in affected subjects has been reported. In particular, the study by Yin et al. [26] assessed the fetal genotype of any locus using maternal plasma, through a novel genotyping algorithm named pseudo tetraploid genotyping (PTG). The mutation in COL1A1 gene, responsible for osteogenesis imperfecta, was successfully detected and a subsequent verification by Sanger sequencing of fetal and parental blood was performed.

#### 3.3.2. Metabolic Disorders

As shown in Table 2, various authors described the application of NIPT for diseases caused by congenital metabolic errors.

You et al. in 2014 [40] and Gupta et al. in 2015 [35] successfully applied NIPD, using haplotype-based and Sanger sequencing methods, respectively, on maple syrup urine disease, caused by mutations in genes BCKDHA, BCKDHB, DBT encoding E1α, E1β, and E2 subunits of enzyme complex, branched-chain alpha-ketoacid dehydrogenase (BCKDH). Deficiency or defect in the enzyme complex causes accumulation of BCAAs and keto-acids and leads to toxicity. In Gupta’s paper, many cases presented in the neonatal period. Prenatal diagnosis was performed in four families.

In 2018, Bijarnia-Mahay presented a study [27] in which NIPT was applied for urea cycle disorders (UCDs). The clinical phenotype is highly variable and is basically characterized by hyperammonemia accompanied therefore by significant mortality and morbidity in infants and children. Of the 123 cases, the majority of them (58%) with 88% on or before day 7 of life, presented in the neonatal period (classical presentation); mortality was high (88%). Such findings stress the importance of a genetic diagnosis of the disease already in the gestational period. The three most observed pathologies were citrullinemia type 1, ornithine transcarbamylase (OTC) deficiency and argininosuccinic aciduria. The overall clinical outcome has shown an overall all-time mortality of 63% (70/110 cases with a known follow-up), and disability in 70% among the survivors. Prenatal diagnosis was performed in 30 pregnancies in 25 families, including one pre-implantation genetic diagnosis.

Among the conditions caused by the accumulation of organic acids, Table 3 shows that NIPD was described only for methylmalonic acidemia/aciduria [2,17]. Specifically, the 2022 paper by Lv et al. [17] relied on trios molecular diagnosis performed in 29 cblC type MMA-affected children and their parents by traditional Sanger sequencing. In the second pregnancy, invasive prenatal diagnosis (IPD) was performed to determine fetal genotypes and, subsequently, NIPT was performed using a novel MMACHC gene-specific cSMART assay. Between NIPT and IPD the concordance ratio was 100%; the sensitivity and specificity were both 100%, further validating the efficacy of the technique.

Only Morsheva et al. reported in 2021 the implementation of NIPD of mitochondrial disorders [22]. This a complex nosological group characterized by various modes of hereditary transmission, depending on the presence of the mutated gene on mitochondrial DNA proper (mtDNA) or nuclear DNA. In this study, carried out on 645 cell-free DNA (cfDNA) samples of pregnant women from different regions of Russia, authors found that, despite the relatively low sequencing depth of unamplified mtDNA from cfDNA samples, the mtDNA analysis in these samples is a valid tool, suitable for screening purposes. In fact, it was possible to analyze effects, frequency and location of mitochondrial variants culled from samples. This procedure led to haplogroup analysis and revealed the most common mitochondrial superclades. Prenatal diagnosis of mitochondrial diseases is very useful because they are diseases characterized by multi-organ involvement and therefore often difficult to clinically diagnose in the growing child [22].

Ye et al., in a study published in 2018 [28], applied a haplotype-based approach for non-invasive prenatal diagnosis of hyperphenylalaninemia (HPA) successfully in 13 families (five fetuses were identified to harbor bi-allelic pathogenic variants, four fetuses were carriers of one heterozygous PAH gene variant, other four fetuses were normal). In this study there was full concordance between NIPD and IPD based on amniotic fluid. In addition, Chen et al., among the pathologies treated in the paper published in 2021 [2], applied NIPT techniques on eight families at risk for phenylketonuria.

#### 3.3.3. Neuromuscular Pathologies

Most of the papers on this group of pathologies concern Duchenne muscular dystrophy [18,20,25,36], a neurodegenerative pathology that brings progressive muscle degeneration until subsequent exitus due to respiratory muscle involvement. The course of the disease is variable among patients and the appearance of the first clinical signs is around 2–5 years of age. In the case papers of Xu, Jang, and Kong [20,25,36] (based on haplotype-based analysis), full agreement between the results of non-invasive and invasive methods was found. In the work of [18], they used a method called relative mutation dosage (RMD)-based approach cell-free DNA barcode-enabled single-molecule test (cfBEST). This technique is easier to perform than the relative haplotype dosage analysis in which the parental haplotypes need to be constructed. Furthermore, the technique based on haplotypes it is not suitable for the diagnosis of de novo mutations or mosaicism in germ cells [18]. Even with the new method, the authors reported full agreement of the results with IPD (one fetus was female and did not carry the familial molecular alteration, three fetuses were carriers, and one was male without the familial mutation).

Only one of the studies [29] was focused on spinal muscular atrophy (SMA), a pathology that causes most of the genetically determined hypotonia in children. This disease is due to mutations in the SMN1 gene or the SMN2 gene that cause a reduction in motor neuron survival. There are five different forms of spinal muscular atrophy (type 0, type 1, type 2, type 3 and type 4). The first three types are very serious and cause premature death of the patient; type 3 and type 4 are milder variants. For selective patients, gene therapy is available, which is still very expensive. In this work, six pregnant SMA carriers and ten healthy pregnant donors were recruited and sequencing data was analyzed by relative haplotype dosage (RHDO). For all patients tested, NIPT results showed a testing specificity and sensitivity of 100%.

#### 3.3.4. Hematologic Disorders

Among hematologic disorders, various papers focused on NIPD of alpha or beta thalassemia [19,24,31,41]. In the study of D’Souza [41], the non-invasive approach gave comparable results to those obtained by the conventional invasive fetal sampling methods in 24 cases, with an accuracy of 80.0%. This result was indicated by the authors as not sufficient for clinical application. In the subsequent works of Yang and Vermeulen [24,31], both based on analysis carried out with haplotype method, a full concordance between the results of the non-invasive and the invasive method emerged (Yang’s work is the only one in which the technique has been evaluated also for alpha thalassemia) [24,31]. The 2021 study by Lv et al. [19] is based on plasma samples collected from 102 pregnant Chinese couples carrying pathogenic HBB gene variants and, retrospectively, used a cSMART assay for fetal genotyping. The pregnancies had been managed with diagnosis by invasive technique. Among these, 99 of 102 fetuses (97%) were correctly genotyped by NIPD assay (sensitivity 100% and specificity 97.26%). These data also support excellent reliability of the non-invasive test for beta thalassemia, probably also for alpha type.

Only one study has addressed the application of NIPD for Hemophilia A and B [42]. Samples were collected from 12 patients and the analysis was conducted with relative mutation dosage approach; the procedure led to the correct identification of mutations on the X chromosome on all samples.

#### 3.3.5. Skin Diseases

Skin diseases caused by the mutation of a single gene or a chromosomal locus are called “genodermatosis” and more than 500 of them have been described. Many of them tend to be chronic and have no definitive cure, significantly impacting the lives of patients. In the study by Ma et al. published in 2013 [45], the authors discussed some ethical issues in NIPD for genetic skin diseases of various severities and in particular for the three diseases: Marie Unna hereditary hypotrichosis (MUHH), familial hidradenitis suppurativa (HS) and harlequin ichthyosis (HI). MUHH is an autosomal dominant form of genetic hair loss in which the patients are born with sparse or absent hair; after entering puberty, their hair will lose progressively. Characteristic features are the absence of eyebrows, eyelashes and body hair and the culprit gene is U2HR. HS is a chronic inflammatory disease of skin follicles, characterized by recurrent skin abscesses, painful sinus tracts with suppuration and hypertrophic scarring in the apocrine gland-bearing area. Typically, HS occurs after puberty and the main triggers are often smoking and obesity. This disease can complicate into squamous cell carcinoma, lymphoedema, fistulae formation and often depression with dramatic impairment of quality of life. This disease is usually inherited in an autosomal dominant pattern and various mutations have been identified in NCSTN, PSENEN and PSEN1 genes in different families. HI, the most severe of the three, is an autosomal recessive hereditary skin disorder with very bad prognosis (survival rate is only 56%) caused by mutations in the ABCA12 gene. The classic phenotype is already visible in the neonate (called ‘harlequin fetus’) and is characterized by dysplastic nose and ears, thick truncal skin plaques with deep fissures, bilateral ectropion and eclabium. Without early treatment, most of the neonates would die soon after birth. Survivors develop poor hair growth, nail deformities, persistent ectropion, gastrointestinal dysfunction, low body height and weight, digital contractures, respiratory infection and intellectual impairment [38]. One case of invasive prenatal diagnosis on amniocytes (with subsequent termination of pregnancy) has been reported in the literature for this condition [46]. Although no cases of families at risk for dermatological diseases in which NIPD was performed have been reported, it is plausible that, improving future analysis protocols, some diseases belonging to the group of genodermatoses will also be included, given the significant impact on the health of the unborn child and his/her quality of life.

#### 3.3.6. Other Conditions (Wilson Disease, Cystic Fibrosis, Non-Syndromic Hearing Loss, Polycystic Kidney Disease, 46XY Sex Development Disorders)

NIPD of non-syndromic hearing loss appears in three studies [2,30,33]. The first two focused exclusively on this condition (caused by mutations in the GJB2, GJB3 and SLC26A4 genes). Chen et al. in 2016 [33] collected a total of 25 plasma samples selected with different fetal NSHL genotypes and retrospectively analyzed by NIPT using a cSMART assay. Concordance with neonatal genotypes was detected in all samples. Han et al., in 2017 [30] recruited 80 pregnant couples carrying known mutations in either the GJB2 or SLC26A4 genes. The results of the analysis, also in this case performed with cSMART assay, led to a correct identification of the genotype in 91.35% of cases; considering the samples with fetal DNA fractions >6%, the sensitivity and specificity of the cSMART assay for correctly diagnosing ARNSHL were 100 and 96.5%, respectively.

Wilson’s disease (WD) is caused by altered copper metabolism with consequent complex clinical presentations characterized by hepatopathy (up to possible cirrhotic evolution) and neurological-psychiatric disorders. Among the clinical signs of greater diagnostic specificity, although not always present, we find the ocular rings of Kaiser-Fleischer. NIPD of this condition has been reported in a single study [37], specifically four families with WD pedigrees were recruited. Using a cSMART assay, the authors retrospectively showed in second pregnancies the concordance of fetal genotypes between non-invasive and invasive methods.

Mutations in the HSD17B3 gene cause 46,XY disorders of sex development (46,XY DSD), pathologies often difficult to identify and which are often confirmed only at older ages, when an affected XY female presents with primary amenorrhea or develops progressive virilization. In these cases, obviously, the ultrasonographic investigation for sex determination is not successful. In the case of De Falco 2021 [23], exome sequencing was performed on the cell free fetal DNA (cffDNA) and a panel of sexual disease genes was used in order to search for a causative variant; the finding of the mutation on the previously mentioned gene (c.645 A > T, p.Glu215Asp) was correct, as well as the invasive investigation on amniotic fluid.

NIPD of cystic fibrosis was reported in the previously cited 2017 study by Vermeulen et al. [31], with good efficacy despite the small number of samples.

Polycystic kidney disease with autosomal recessive transmission was previously cited in the 2021 paper by Chen et al. [2]. Although only one at-risk family was recruited, the pathogenic variant was successfully detected.

### 3.4. Causes of False Positives and False Negatives in cffDNA Test

Another important point to discuss is the possibility of false positives (FP) and false negatives (FN) of NIPT test with cffDNA. FP occurs in 0.3% of cases and, among the various causes, we find placental mosaicism (PM), abortive twins and maternal mosaicism. PM is due to the fact that the first source of cff-DNA in the maternal circulation is basically the placenta (particularly syncytiotrophoblasts). The phenomenon of placental mosaicism occurs in 1 to 2% of pregnancies and is more likely to occur with Turner syndrome (XO) and triploidy 13 [54,55]. Another cause of false positives may be a previous abortion of a twin (vanishing twin); in that case, the placenta of the dead fetus, which continues to send DNA into the maternal circulation weeks later, creates the interference. Another important cause is maternal mosaicism. In fact, especially in older women, maternal cells that lost an X chromosome increase and can alter test results. There are also women who, despite a normal phenotype, have hidden chromosomal alterations (e.g., 47, XXX). Another FP cause may be maternal cancer. In fact, tumor free DNA can be introduced into the maternal circulation in women with a malignancy in pregnancy and contribute to the total cfDNA [56].

FN are less common than FP and account for 0.01%; the main cause is certainly the reduced availability of cfDNA. In fact, sometimes cfDNA is sufficient for the test but it counts at the lower limit (often less than 4%). Without a sufficient number of DNA segments, no reliable comparison with the normal human reference fragments can be made, and that can result in FN being detected. This phenomenon is possible when the mother has used low molecular weight heparin (LMWH) such as enoxaparin or in case of obesity.

## 4. Discussion

In this review we have reported all the papers in which the use of non-invasive prenatal diagnosis techniques for chromosomal and monogenic transmission diseases has been described. Regarding chromosomal aneuploidies, the high median detection rate for the three most common trisomies (21,18,13) certainly justifies the use of the test as a screening tool for these diseases. The efficacy was reported as comparable between low-risk and high-risk pregnancies and we reiterated how important the value of the fetal fraction analyzed is to validate the effectiveness of the test. There is still disagreement among the different authors as to whether the efficacy of the test is comparable between singleton and twin pregnancies. From the data examined, there seems to be a good performance of the test in twin pregnancies for Down syndrome whereas, for 18 and 13 Trisomy, given the lower incidence, there are still few works able to express certainty. Given the effectiveness of NIPT as a screening test, several authors have investigated the possibility of using it as a real diagnostic tool. This, consequently, would lead to a reduced use of invasive methods and related complications. Several authors have expressed themselves in favor of this hypothesis, mainly because of the high positive predictive value of the test, especially for trisomy 21. It is therefore quite plausible that, in the near future, we will talk about NIPT as a diagnostic tool for Down syndrome. Furthermore, in one of the papers the authors reported that, for certain risk couples identified by the combined test of the first trimester, NIPT has equal sensitivity and even superior specificity if compared to invasive methods. It is obviously necessary that further future studies validate this hypothesis. Although various authors reported the superiority of NIPT if compared to the combined first trimester test, in future screening protocols it is likely that, in order to further increase the diagnostic accuracy, NIPT will be associated with ultrasonographic examination with nuchal translucency research.

As for diseases with monogenic transmission, in this paper we have illustrated the various studies in which the use of NIPD techniques has been described, separated according to the nosological category. It is worth noting that for all the groups treated (endocrinological, skeletal, hematological, neuromuscular, metabolic, mitochondrial diseases, etc.) the authors have reported very high rates of correspondence between the results of the analysis obtained with non-invasive method and those obtained with invasive method (carried out as a confirmation method in almost all cases described). Although there are more advanced methods of analysis than others, the diagnostic accuracy of these tests is reportedly very high, regardless of the technique used. In any case, most of the data concern analyses conducted with haplotype-based approaches. It should be emphasized that all authors, even for monogenic disorders, have decreed reliable analysis in samples with adequate fetal fraction, preferably greater than 6%.

What has been said is extremely important, first of all, because it fully justifies the use of NIPD techniques as a screening test also for a wide variety of diseases with monogenic transmission, an option still not present in all national health care systems. Another aspect to emphasize is that, given the high detection performance, it is possible that in a near future NIPT will become not only a screening tool, followed by the validation of invasive test, but a real diagnostic tool, as mentioned earlier for Down syndrome. Certainly, what has been said is more likely for diseases with a greater number of dedicated studies, specifically congenital adrenal hyperplasia, Duchenne muscular dystrophy, beta-thalassemia and some metabolic diseases such as methylmalonic aciduria and maple syrup urine disease.

In contrast, for diseases caused by chromosomal microdeletions/duplications, the results of NIPT were not encouraging, except in part for Di George syndrome. A major issue with CNV lies in the fact that many alterations detected, in the case of a shallow NGS sequencing, risk not being confirmed and are therefore false positives. Instead, using NGS sequencing of adequate depth could provide an adequate method to detect CNVs with relatively advantageous costs [48].

As already mentioned in the previous paragraphs, for some of these diseases an early prenatal diagnosis, made through non-invasive methods, would lead to lower morbidity and mortality rates in the first months or even days of life. This is especially true for urea cycle disorders that occur in neonatal age (in more than half of cases) and are often followed by the child’s death. For other diseases, specifically for congenital adrenal hyperplasia, NIPD represents the only tool capable of discriminating the presence of mutations in time to start therapy with dexamethasone, avoiding exposure to treatment of unaffected fetuses but in pregnancies at risk.

For other mentioned diseases, an early diagnosis is a valuable tool to start therapeutic measures already in the first months of extrauterine life and prevent in time the worst complications. Further studies are certainly required to confirm what has been said and provide further information on NIPD for pathologies not yet reported.

Another important issue concerns the accessibility of the test by the population. A study by the Dutch NIPT consortium found that NIPT uptake in socioeconomically disadvantaged neighborhoods was significantly lower compared to all other neighborhoods (20.3 vs. 47.6%). Such a difference in NIPT uptake was reportedly smaller for the youngest maternal age-group (≤25 years). However, this variation in uptake could create disparities that would undermine the goals of a national fetal aneuploidy screening program [47].

In essence, although NIPT does seem to be a very promising method of diagnosis for several diseases the use of such a test for conditions other than trisomies is still controversial. An interesting study by Christiaens et al., published in 2021, reported a debate on the extensive use of NIPT. Participants were invited to express themselves “in favour” or “against”. In the first vote the results were 65% and 35%, respectively. A discussion then followed on the pros and cons of the test, after which respondents were asked to vote again. The new percentages were 41% and 59%. Such a discrepancy seems to point to the need to further raise awareness before NIPT can be extended to test for conditions other than common aneuploidies, although it could be at least theoretically very valid and reliable [57] for various different applications.

### Legal and Ethical Implications Call for Caution

The innovative nature of NIPT makes it necessary to exercise caution in the way such services are offered and delivered, since medicolegal implications may arise and involve doctors and facilities. Negligence is arguably the most common grounds on which litigation may arise. “Unreasonable risk” resulting from the breach of legal duties rather widespread in all medical malpractice-related litigation, and prenatal testing is no exception [58]. Negligence and malpractice allegations may be linked to the failure on the part of healthcare professionals to provide services that meet the standards of reasonable professional care in force when the intervention was implement. As a consequence, claimant patients could point to damage having been caused that would not otherwise have occurred as a direct result of such non-compliance [59].

Malpractice charges linked to prenatal genetic testing procedures can be brought on various grounds. Physicians or genetic counselors can for instance be charged with negligence in the provision of genetic counseling, e.g., failing to thoroughly inform patients as to the potential reproductive risk associated with carrier status or age, denying requests to execute invasive procedures or keep from patients relevant information concerning the need or availability of such procedures. When discharging a thorough informed consent process, doctors have a professional duty to expound upon, and start a discussion about, risks, benefits, and possible alternatives to a given procedure. Explaining and discussing possible alternatives constitutes a fundamental element of the disclosure process; patients may in fact be unable to evaluate the risks in abstract terms, and would therefore need to rely on a frame of comparison in order to make a truly informed decision. Litigation may also arise from laboratory negligence allegations, e.g., genetic counseling grounded in misconstrued laboratory results leading patients to make decisions that they would not have made if they had been given factual and thorough information. Those may even include the choice to bring the pregnancy to term. Such instances are known as “wrongful birth” or “wrongful life” cases [60]. Such dynamics involving incomplete or misinterpreted laboratory results may also lead to lawsuits where claimants argue that they would not have aborted a fetus had they been correctly diagnosed or received appropriate counseling as to the risks of terminating a pregnancy without further confirmatory test results. Such instances are referred to as “wrongful abortion” lawsuits. Both wrongful birth and wrongful abortion entail major legal and ethical complexities that cannot be discounted [61,62]. Scholars should therefore set out to start a wide-ranging discussion as to the results and implications, and how they can be interpreted from the perspective of previous studies and of the working hypotheses. The findings and their implications should be discussed in the broadest context possible, getting all stakeholders involved.

## 5. Conclusions

Our review summarizes data regarding the non-invasive prenatal diagnosis through fetal cell-free DNA. The screening value of this tool has been asserted by numerous research sources, which reaffirms the important role of a non-invasive approach in the first trimester of pregnancy. The scientific community needs to focus on providing new insights on the most accurate diagnostic definitions, aimed at specific conditions, of this prenatal testing technique, by taking into account the best standards for ensuring clinical, ethical and legal viability.

## Figures and Tables

**Table 1:** Non-invasive prenatal diagnosis of chromosomal aneuploidies studies included in the review.

Authors (et al.); Year	Number of Patients	Type of Study	21 Trisomy Detection Rate (%)	18 Trisomy Detection Rate (%)	13 Trisomy Detection Rate (%)
Judah 2021 [4]	1442	Cohort and Review	99	92,8	94,7
Borth 2021 [5]	13,607	Cohort	98.89	99.99	99.99
Serapinas 2020 [6]	850	Cohort	100	100	/
Gil 2019 [7]	997	Cohort	98.2	88.9	66.7
Miltoft 2018 [8]	597	Cohort	100	100	100
Gil 2017 [9]	661,473	Review and meta-analysis	99.7	97.9	99
Taylor-Phillips 2016 [10]	Not specified	Review	99.3	97.4	97.4
Zhang 2015 [11]	112,669	Cohort	99.17	98.24	100
Porreco 2014 [12]	3340	Cohort	100	92.3	87.5
Lau 2014 [13]	1982	Cohort	100	100	100
Stumm 2014 [14]	485	Cohort	95.2	100	100
Liang 2013 [15]	435	Cohort	100	100	100
Median detection rate			99.50	99.12	99.99

**Table 2:** Non-invasive prenatal diagnosis of copy number variation pathologies in studies included in the review. Most of these anomalies, and therefore of the pathologies related to them (in the case of pathogenetic variants), arise sporadically; cases of inheritance are limited.

Authors (et al.); Year	Patients	Pathologies	Genes
Van der Meij 2021 [47]	15,562	Not specified	Not specified
Songchan 2021 [48]	11,903	Not specified	Alterations in the number and/or fractions of chromosomes 1, 2, 3, 5, 9, 13, 16, 18
Yunsheng 2021 [49]	18,516	Not specified	Alterations in the number and/or fractions of chromosomes 1, 4, 9, 11, 12, 13, 14, 15, 20
Liang 2019 [50]	94,085	Aneuploidies, DiGeorge, 22q11.22 microduplication, PW/Angelman, Cri du Chat and other microdeletion/duplication syndromes	Alterations in the number and/or fractions of chromosomes 22, 21, 18, 15, 13, 5.
Van der Meij 2019 [51]	73,239	PW and other non-specified conditions	Alterations in the number and/or fractions of chromosomes 9, 12, 15 and others
Kaseniit 2018 [52]	87,255	Not specified	Alterations in the number and/or fractions of chromosomes 1, 4, 5, 13, 15, 22
Petersen 2017 [53]	712	Aneuploidies, DiGeorge, 22q11.22 microduplication, PW/Angelman, Cri du Chat and other microdeletion/duplication syndromes	Alterations in the number and/or fractions of chromosomes 22, 21, 18, 15, 13, 5

**Table 3:** Non-invasive prenatal diagnosis of monogenic transmission diseases and chromosomal microdeletion/duplication studies included in this review.

Authors (et al.)	Year of Publication	Samples	Pathologies	Genes	Hereditary Transmission
Lv [17]	2022	29	Methylmalonic aciduria cblC type	MMACHC	AR
Zhao [18]	2021	5	Duchenne muscular dystrophy	DMD	X-Linked
Chen [2]	2021	40	Methylmalonic acidemia/aciduria, phenylketonuria, alfa/beta-thalassemia, ARPKD, DFNB1A	MMACHC, PAH, HBA, HBB, PKHD1, GJB2	AR
Lv [19]	2021	102	Beta-thalassemia	HBB	AR
Kong [20]	2021	21	Duchenne muscular dystrophy	DMD	X-Linked
Wang [21]	2021	59	Skeletal dysplasia	FGFR2, FGFR3, COL1A1, COL1A2 and COL2A1	AD
Morshneva [22]	2021	645	Mitochondrial disorders	mtDNA variants	Variable
De Falco [23]	2021	1	46 XY disorders of sex development	HSD17B3	Variable
Yang [24]	2020	8	Alpha and beta-thalassemia	HBA and HBB	AR
Jang [25]	2018	5	Duchenne muscular dystrophy	DMD	X-Linked
Yin [26]	2018	1	Osteogenesis imperfecta	COL1A1	AD
Bijarnia-Mahay [27]	2018	123	Urea cycle disorders	ASS1, ASL, OTC, ARG1, CPS1, NAGS, SLC25A13, SLC7A7	X-Linked (OTC) and AR
Ye [28]	2018	13	Hyperphenylalaninemia	PAH	AR
Parks [29]	2017	6	Spinal muscular atrophy	SMN1	AR
Han [30]	2017	80	Non-syndromic hearing loss	GJB2 and SLC26A4	AR
Vermeulen [31]	2017	18	Cystic fibrosis, congenital adrenal hyperplasia and beta-thalassemia	CFTR, CYP21A2, and HBB	AR
Ma [32]	2017	14	Congenital adrenal hyperplasia	CYP21A2	AR
Chen [33]	2016	25	Non-syndromic hearing loss	GJB2, GJB3 and SLC26A4	AR
Dan [34]	2016	3	Thanatophoric dysplasia, osteogenesis imperfecta type II, and achondroplasia	FGFR3, COL1A1 and COL2A2	AD
Gupta [35]	2015	24	Maple syrup urine disease	BCKDHA, BCKDHB, DBT	AR
Xu [36]	2015	8	Duchenne muscular dystrophy	DMD	X-Linked
Lv [37]	2015	4	Wilson disease	ATP7B	AR
Ma [38]	2014	1	Congenital adrenal hyperplasia	CYP21A2	AR
New [39]	2014	14	Congenital adrenal hyperplasia	CYP21A2	AR
You [40]	2014	1	Maple syrup urine disease	BCKDHA	AR
D’souza [41]	2013	30	Beta-thalassemia	HBB	AR
Tsui [42]	2011	12	Hemophilia A and B	F8, F9	X-Linked

## Data Availability

Not applicable.
